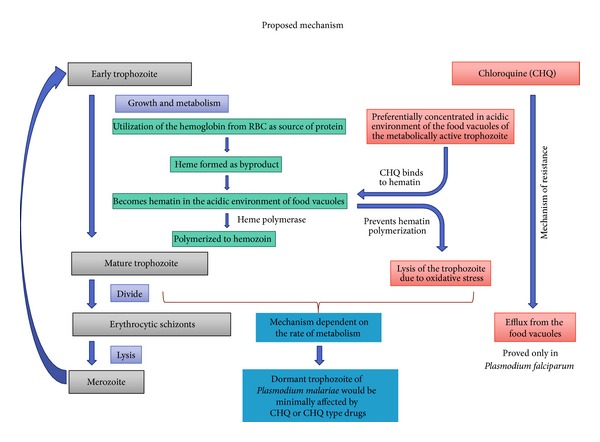# Recrudescence of *Plasmodium malariae* after Quinine

**DOI:** 10.1155/2014/590265

**Published:** 2014-02-12

**Authors:** Irfanali R. Kugasia, Farhana K. Polara, Hussein Assallum

**Affiliations:** ^1^Lincoln Medical and Mental Health Center, 234 E. 149th Street, Bronx, NY 10451, USA; ^2^B. J. Medical College, Asarwa, Ahmedabad, Gujarat 380016, India

## Abstract

*Plasmodium malariae* causes uncommon benign malaria found in the malaria endemic regions mostly of Sub-Saharan Africa. As *Plasmodium malariae* does not have a continued liver stage in humans the only way to have reinfection without reexposure is through recrudescence. However, reports of its recrudescence after antimalarials are rare with only a handful of case reports in the literature. Research in this field to date has not been able to establish definitively an emergence of resistance in *Plasmodium malariae* to commonly used antimalarials. In the presented case, patient had a recrudescence of *P. malariae* after full treatment with quinine and clindamycin. This recrudescence was treated with full course of chloroquine with clearance of parasite from blood immediately after treatment and at two months' follow up. The recrudescence in this case cannot be explained by mechanisms explained in prior articles. We propose that the indolence of some of the *Plasmodium malariae* trophozoites in the blood can shield them from the effect of the toxic effects of antimalarials and enable them to produce recrudescence later. However, when recrudescence happens, this should not be considered a case of development of resistance and a course of chloroquine should be considered.

## 1. Introduction

*Plasmodium malariae* infections are infrequently found in the malaria endemic regions with majority of them reported from Sub-Saharan Africa and Southeast Asia. There they are commonly found as mixed infections with *Plasmodium falciparum* [1]. Unlike *Plasmodium vivax* and *Plasmodium ovale*, *Plasmodium malariae* is not known to have continued liver cycle with hypnozoites. The only way to have a reinfection without reexposure is from its preexisting erythrocytic forms; this is known as recrudescence. These erythrocytic forms of *Plasmodium malariae* are known to be the most indolent of all the infective plasmodium species with infections observed decades after exposure. Recrudescence of *P. malariae* is common if the primary episode of infection goes untreated [1, 2]. However, only a handful of cases have reported recrudescence of *Plasmodium malariae* even after treatment with different antimalarials [2, 3]. Till date no conclusive evidence is presented in the literature regarding emergence of resistance in *Plasmodium malariae* that can explain recrudescence in these cases [4–7].

## 2. Case

The case is of a 65-year-old migrant from Sierra Leone who has been living in the United States for more than a decade. The patient has had history of multiple episodes of malaria infection in childhood but no reported episode after emigrating to the United States. The patient had last been to the malaria endemic region of Sierra Leone in 2009 and denied any sickness during her trip or immediately after returning to the United States. She had her first episode of malaria in August of 2010 during which she was treated with full course of quinine and clindamycin. Her blood smears examined for parasites were reported as positive for *Plasmodium falciparum* trophozoites with very low parasite titer. The patient was readmitted in September of 2012 with complaint of recurrent fever and chills. She had no interval history of travel to any malaria endemic region. Blood smears obtained on the second hospitalization were reported showing *Plasmodium malariae* trophozoites. The patient's peripheral blood smears from the current and prior hospitalization were reviewed by the hospital's chief pathologist to answer why two different malariae species were identified on the two occasions without any evidence of reexposure. The final pathology report stated that the malaria parasite seen on both occasions was that of *Plasmodium malariae* and the initial reports of the parasite being *Plasmodium falciparum* were flawed. After this report the patient was treated with full course of chloroquine with resolution of fever and parasitemia. At 2-month followup a blood smear was obtained which did not show any parasites in her blood.

## 3. Discussion

A number of theories have been proposed to explain the mechanism of reinfection with *P. malariae* after initial antimalarial treatment [2, 3]. Firstly, it might not be a recrudescence but a case of reexposure resulting in reinfection. Secondly, it could be from recrudescence as a result of patient being noncompliant with medications course. Thirdly, the recrudescence could be because of development of resistance in *P. malariae* to the antimalarial medications. Fourthly, it could be a recurrence from prolonged preerythrocytic liver cycle which was not affected by the initial course of antimalarial medications. Lastly, it could be from subtherapeutic plasma level of antimalarials and concomitant immunity. Bearing the last one none of the other theories could explain the reinfection observed in this case as explained here.

1. Reexposure was not possible in this patient as she has not travelled out of the United States in the interval between her hospitalizations and there are zero cases of malaria transmission reported in the United States. All malaria cases reported in the United States were imported [8].
2. The patient confirmed that she had completed the full course of antimalarials prescribed to her, which were quinine and clindamycin.
3. Development of resistance in *P. malariae* to antimalarials is a compelling theory. Isolated cases of recrudescence of *Plasmodium malariae* have been reported after almost all types of antimalarial medications like quinine, chloroquine, mefloquine, and artesunates [2, 3, 9]. Review of the literature showed only one study reporting resistance of *Plasmodium malariae* to chloroquine in 2 cases which failed to clear the parasite from blood during the 4 days of treatment [7]. However, it has been shown that trophozoites of *Plasmodium malariae* take longer time to clear up from the blood compared to other *Plasmodium* species. In some cases it was up to 16 days [5]. Also multiple other studies *in vivo* and *in vitro* have proved the sensitivity of *P. malariae* to chloroquine [4, 6, 10]. Hence there is no compelling evidence for development of resistance and both WHO and CDC recommend treatment of *Plasmodium malariae* with chloroquine or quinine.
4. Reinfection from preerythrocytic forms is not plausible in this case as the episodes of infection were almost 1 year and 3 years from the last presumed exposure to the parasites. *Plasmodium malariae* is not known to have continuous reproduction of preerythrocytic forms [1, 3].
5. Subtherapeutic plasma level could be a possible explanation for treatment failure. Studies have shown subtherapeutic plasma concentration after the regular chloroquine course and plasma levels below MIC levels have been implicated with slow clearance of parasitemia [11]. However this theory would not explain why a repeat course of same medications would be able to treat a reinfection.

The recrudescence in this case and other cases reported before can be attributed to the indolence of the erythrocytic forms of *Plasmodium malariae*. It is well known that asexual erythrocytic forms of *Plasmodium malariae* are very indolent and can undergo very slow metabolism and growth for decades [1].  Figure 1 summarizes how indolence of *P. malariae* can shield it from the toxic effects of chloroquine and hence provide *P. malariae* an inherent resistance to its action. Though this mechanism is presented for chloroquine, it can be extrapolated to other antimalarial drugs like quinine and mefloquine which are considered to have similar mechanism of action. The indolent erythrocytic schizonts of *P. malariae* have a low rate of metabolism and hence reduced size and acidity of the food vacuoles which is dependent on their metabolic rates. The acidity in the food vacuoles is important in concentrating the chloroquine and quinines in the food vacuole because of their basic nature. Low metabolic rate can shield these indolent schizonts to survive the initial course of antibiotics. These indolent schizonts then persist in low grade erythrocytic cycle of propagation to produce recrudescence later.

In conclusion we propose that recrudescence of *P. malariae* should not be always counted as a case of resistance to antimalarials and a repeat course of chloroquine should be considered.

## 4. Critique

A major critique to this case is that no other confirmatory tests like PCR or antigen testing were obtained. On initial presentation patient could have had a mixed infection which is commonly found in Sub-Saharan Africa. However, in a mixed infection it is difficult to identify *P. malariae* on a blood smear rather than *P. falciparum* due to low titres of *P. malariae*'s trophozoites in blood. A PCR analysis of patient's blood sample from initial hospitalization could have confirmed if it was a mixed infection or only a *P. malariae* infection. This was not possible in this case due to lack of blood samples from prior hospitalization of the patient. As a result, all the slides from both hospitalizations were thoroughly examined by the pathologist who reported that only *P. malariae* parasites were seen on all ten slides.

## Figures and Tables

**Figure 1:**